# Association of Abnormal Findings on Neonatal Cranial Ultrasound With Neurobehavior at Neonatal Intensive Care Unit Discharge in Infants Born Before 30 Weeks’ Gestation

**DOI:** 10.1001/jamanetworkopen.2022.6561

**Published:** 2022-04-08

**Authors:** Jennifer Helderman, T. Michael O’Shea, Lynne Dansereau, Jennifer Check, Julie A. Hofheimer, Lynne M. Smith, Elisabeth McGowan, Charles R. Neal, Brian S. Carter, Steven L. Pastyrnak, Bradford Betz, Joseph Junewick, Heather L. Borders, Sheri A. DellaGrotta, Barry M. Lester

**Affiliations:** 1 Department of Pediatrics, Wake Forest School of Medicine, Winston Salem, North Carolina; 2 Department of Pediatrics/Neonatal-Perinatal Medicine, University of North Carolina School of Medicine, Chapel Hill; 3 Brown Center for Children at Risk, Women and Infants Hospital of Rhode Island, Providence; 4 Department of Pediatrics, Lundquist Institute at Harbor-UCLA, Torrance, California; 5 Department of Pediatrics, Brown Alpert Medical School and Women and Infants Hospital, Providence, Rhode Island; 6 Department of Pediatrics, University of Hawaii John A Burns School of Medicine, Honolulu; 7 Department of Pediatrics–Neonatology, Children’s Mercy Hospital, Kansas City, Missouri; 8 Department of Pediatrics, Spectrum Health–Helen DeVos Hospital, Grand Rapids, Michigan; 9 Department of Pediatric Radiology, Spectrum Health–Helen DeVos Hospital, Grand Rapids, Michigan; 10 Department of Diagnostic Radiology, Spectrum Health–Helen DeVos Hospital, Grand Rapids, Michigan; 11 Intercity Radiology, Bozeman, Montana; 12 Department of Psychiatry, Brown Alpert Medical School, Providence, Rhode Island

## Abstract

**Importance:**

Cranial ultrasound (CUS) findings are routinely used to identify preterm infants at risk for impaired neurodevelopment, and neurobehavioral examinations provide information about early brain function. The associations of abnormal findings on early and late CUS with neurobehavior at neonatal intensive care unit (NICU) discharge have not been reported.

**Objective:**

To examine the associations between early and late CUS findings and infant neurobehavior at NICU discharge.

**Design, Setting, and Participants:**

This prospective cohort study included infants enrolled in the Neonatal Neurobehavior and Outcomes in Very Preterm Infants Study between April 2014 and June 2016. Infants born before 30 weeks’ gestational age were included. Exclusion criteria were maternal age younger than 18 years, maternal cognitive impairment, maternal inability to read or speak English or Spanish, maternal death, and major congenital anomalies. Overall, 704 infants were enrolled. The study was conducted at 9 university-affiliated NICUs in Providence, Rhode Island; Grand Rapids, Michigan; Kansas City, Missouri; Honolulu, Hawaii; Winston-Salem, North Carolina; and Torrance and Long Beach, California. Data were analyzed from September 2019 to September 2021.

**Exposures:**

Early CUS was performed at 3 to 14 days after birth and late CUS at 36 weeks’ postmenstrual age or NICU discharge. Abnormal findings were identified by consensus of standardized radiologists’ readings.

**Main Outcomes and Measures:**

Neurobehavioral examination was performed using the NICU Network Neurobehavioral Scale (NNNS).

**Results:**

Among the 704 infants enrolled, 675 had both CUS and NNNS data (135 [20.0%] Black; 368 [54.5%] minority race or ethnicity; 339 [50.2%] White; 376 [55.7%] male; mean [SD] postmenstrual age, 27.0 [1.9] weeks). After covariate adjustment, lower attention (adjusted mean difference, −0.346; 95% CI, −0.609 to −0.083), hypotonicity (mean difference, 0.358; 95% CI, 0.055 to 0.662), and poorer quality of movement (mean difference, −0.344; 95% CI, −0.572 to −0.116) were observed in infants with white matter damage (WMD). Lower attention (mean difference, −0.233; 95% CI, −0.423 to −0.044) and hypotonicity (mean difference, 0.240; 95% CI, 0.014 to 0.465) were observed in infants with early CUS lesions.

**Conclusions and Relevance:**

In this cohort study of preterm infants, certain early CUS lesions were associated with hypotonicity and lower attention around term-equivalent age. WMD was associated with poor attention, hypotonicity, and poor quality of movement. Infants with these CUS lesions might benefit from targeted interventions to improve neurobehavioral outcomes during their NICU hospitalization.

## Key Points

**Question:**

What is the association between neonatal cranial ultrasound findings and neurobehavioral examination at term-adjusted age?

**Findings:**

In this cohort study of 675 infants born before 30 weeks’ gestation, abnormal findings on cranial ultrasound were associated with decreased tone, poor regulation of attention, and movement outcomes as the infants matured to term-adjusted age.

**Meaning:**

Among very preterm infants, abnormal findings on cranial ultrasound identifiable in the first 14 postnatal days were associated with neurobehavior outcomes at or near term-equivalent age and could be used to help counsel and educate parents as well as inform treatment strategies for therapy service in the neonatal intensive care unit and after discharge.

## Teaser

This cohort study examines the associations between early and late cranial ultrasound findings and infant neurobehavior at neonatal intensive care unit discharge.

## Introduction

Worldwide each year, approximately 15 million infants, and 10% of US births, are born preterm, defined as delivery prior to 37 weeks’ gestation.^1^ Of those born very preterm, ie, before 32 weeks, moderate to severe neurodevelopmental impairments (NDI) develop in approximately 17%.^2^ This high rate of impairment could be addressed by early detection in at-risk infants and developmentally supportive interventions applied at the earliest point. However, during the first several postnatal months, identifying subsets of very preterm infants who are at increased risk for developmental impairment is challenging.

Neonatal cranial ultrasound (CUS) imaging is used routinely in the neonatal intensive care unit (NICU) to identify brain abnormalities associated with cerebral palsy and other neurodevelopmental impairments.^3,4^ Specific abnormalities associated with impairments include ventriculomegaly (VM), echolucency, bilateral cerebellar hemorrhage, intraventricular hemorrhage (IVH), and germinal matrix hemorrhage (GMH; also referred to as subependymal hemorrhage).^3,5,6^ As infants become medically and physiologically stable over the first several postnatal months, assessments of neurobehavior at term-equivalent age (37-42 postmenstrual weeks) provide valuable information about neuromotor, social, and stress-coping regulatory functions that are associated with prenatal and neonatal medical conditions^7,8,9,10,11,12^ and epigenetic processes.^13^ Furthermore, a standardized assessment of neurobehavior, the Neonatal Network Neurobehavioral Scale (NNNS) has been shown to predict NDI at 2 to 7 years in samples of very preterm newborns as well as infants with varied risk factors.^14,15,16,17,18^

The association between abnormal findings on CUS and findings on the NNNS examination prior to NICU discharge has not been previously studied. The purpose of this study was to evaluate the extent to which CUS findings are associated with NNNS scores at NICU discharge, toward the goal of identifying early neurobehavioral risk indicators that can inform targeted interventions in the NICU and after discharge.

## Methods

### Study Population

The Neonatal Neurobehavior and Outcomes in Very Preterm Infants (NOVI) study was conducted at 9 university-affiliated NICUs in Providence, Rhode Island; Grand Rapids, Michigan; Kansas City, Missouri; Honolulu, Hawaii; Winston-Salem, North Carolina; and Torrance and Long Beach, California; with neonatal enrollment and data collection from April 2014 through June 2016. Enrollment and consent procedures for this study were approved by the institutional review boards of all participating institutions. This study followed the Strengthening the Reporting of Observational Studies in Epidemiology (STROBE) reporting guideline for cohort studies.

Eligibility was determined based on the following inclusion criteria: (1) birth before 30 weeks’ postmenstrual age (PMA); (2) parental ability to read and speak English or Spanish; (3) residence within 3 hours of the NICU and follow-up clinic.^11^ PMA at birth was estimated using the approach used in the Extremely Low Gestational Age Newborns (ELGAN) Study.^19^

Exclusion criteria included maternal age younger than 18 years, maternal cognitive impairment, and maternal death. Infants were excluded from eligibility for major congenital anomalies (defined as ≥1 structural defects present at birth that required significant surgical and/or medical intervention) and NICU death.^20,21^ Parents of eligible infants were invited to participate in the study when the attending neonatologists regarded survival to discharge as likely. Informed consent for participation was obtained from the parents.

A total of 704 infants were enrolled and survived to discharge. The present study sample included infants with complete neurobehavioral and CUS data. A total of 679 infants were assessed with the NNNS, and 675 infants had at least 1 CUS prior to NICU discharge, 658 of whom had early scans (day of life, 3-14), and 675 had both early and late scans (at 36 weeks’ PMA or NICU discharge) indicating white matter damage (WMD) (Figure).

**Figure.**
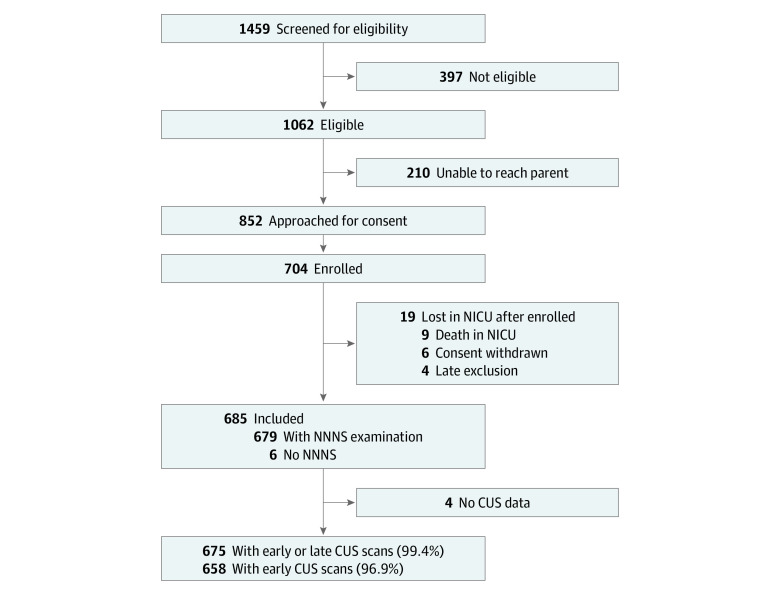
Study Flowchart CUS indicates cranial ultrasound; NICU, neonatal intensive care unit; and NNNS, NICU Network Neurobehavioral Scale.

### Measures

#### Medical Data

Medical records for intrapartum and neonatal hospitalization were reviewed by research coordinators with oversight by study neonatologists at each site to collect information about specific conditions and interventions associated with respiratory, central nervous system (CNS), kidney, cardiac, genetic, hematologic characteristics and function, infections and severe retinopathy of prematurity (ROP). Data were entered using the Vermont Oxford Network (VON) diagnostic definitions and guidelines.^21^

#### CUS

CUS was performed as an aspect of clinical care. Most infants underwent at least 2 ultrasound scans; first, within 14 days of birth (ie, early scans) and again at or about 36 weeks’ PMA or around the time of NICU discharge (ie, late scans).^22^ At all sites, ultrasounds were performed using high-frequency transducers and included the 6 standard quasi coronal views and 5 parasagittal views using the anterior fontanel as the sonographic window. For each study participant, an early and a late ultrasound (as defined previously) were read initially as part of clinical care and were reread by 1 of 2 radiologists (H.B. and J.J.) who had been trained using ELGAN study criteria to characterize the types and locations of abnormal findings with detailed specifications for severity.^23^ If the initial interpretation of the ultrasound findings disagreed with the second interpretation with regard to the presence of a major abnormality (defined as cerebellar hemorrhage, periventricular leukomalacia [PVL], IVH, parenchymal echolucency or echodensity, and moderate-to-severe VM), a third interpretation was provided by 1 of the radiologists who had been trained using ELGAN Study criteria as a tiebreaker.

WMD was defined as presence of PVL, moderate to severe VM, or parenchymal echolucency present on any early or late CUS image. Early CUS lesions included GMH, moderate to severe VM, intraventricular hemorrhage (IVH), and parenchymal echodensity (PED).

#### Neonatal Neurobehavioral Assessments at NICU Discharge

Neonatal neurobehavior was assessed using the NICU Network Neurobehavioral Scale (NNNS). The NNNS is a 20- to 30-minute standardized procedure that includes measures of active and passive tone, primitive reflexes, items that reflect physical maturity, social and behavioral functioning including visual and auditory tracking, cuddling and soothability, and a checklist of stress signs organized by organ system.^9^ The NNNS was administered during the week of NICU discharge (±3 days) by experienced and previously certified examiners following standardized multisite training procedures.^24^ The examination was conducted prior to the next scheduled feeding or routine care to maximize alertness and avoid disrupting NICU routines that facilitate sleep patterns. Examiners were blinded to CUS and other medical complications. Individual items were converted to 13 previously validated summary scores.^14,25^ Detailed findings regarding prenatal and perinatal correlates of NNNS summary scores and risk profiles for NOVI infants were published earlier.^11,13,26^ Habituation was omitted from analyses, as 50% of infants were not in the appropriate state during initiation of examination. The remaining 12 summary scores were examined.

### Statistical Analysis

Unadjusted associations and mean differences of NNNS summary scores and neonatal characteristics by WMD and early CUS lesions were examined by χ^2^ tests or analysis of variance where appropriate. Statistical significance was set at *P* < .05. The association of WMD and early CUS lesions with infant neurodevelopment as measured by the NNNS was evaluated using generalized estimating equations (GEE) regression models specifying unstructured covariance and a normal distribution of the dependent variable to test the difference between the mean NNNS summary scores for infants with evidence of WMD and early CUS lesions for each NNNS summary score adjusting for neonatal characteristics. For these comparisons, a Bonferroni correction (*P* < .025) was applied to the *P* value to adjust for multiple comparisons. We adjusted for characteristics that previously have been reported in the literature as being associated with various neurodevelopmental outcomes and abnormal findings on CUS. The GEE models accounted for site and multiple births (with infants nested within families). Model covariates were chosen a priori based on previous literature and included infant sex, minority race and ethnicity (American Indian, Asian, Black, Hispanic, Native Hawaiian, Pacific Islander, or other specified by mother), chronic lung disease (CLD), severe ROP, sepsis, outborn vs inborn status, PMA at birth, and PMA at NNNS exam. All analyses were conducted with SAS version 9.4 (SAS Institute). Data were analyzed from September 2019 to September 2021.

## Results

A total of 704 infants were enrolled in the NOVI study, and of those, 675 (135 [20.0%] Black; 368 [54.5%] minority race or ethnicity; 339 [50.2%] White) had CUS data. More than half of the infants were male (376 [55.7%]), and the mean (SD) PMA at birth was 27.0 (1.9) weeks (Table 1). A total of 658 infants had an early CUS performed at approximately 1 week of life (mean [SD], 7.5 [3.0] days). For 297 CUS readings (44%), a third radiologist also interpreted the scan to provide a tiebreaker with regard to the presence of a major abnormality. Primary readers agreed 69.3% of the time (456 scans) for early CUS readings and 70.5% of the time (476 scans) related to primary findings, including echodensity.

**Table 1.** Neonatal Characteristics by White Matter Damage and Early CUS Lesions

Characteristic	Infants, No. (%)		OR (95% CI)	Infants, No. (%)		OR (95% CI)
	White matter damage (n = 76)^a^	No white matter damage (n = 599)		Early CUS lesions (n = 156)^b^	No early CUS lesions (n = 502)	
Minority race or ethnicity^c^	40 (53.3)	346 (58.1)	0.78 (0.48 to 1.27)	84 (53.8)	293 (58.7)	0.82 (0.57 to 1.18)
Poverty, public assistance, Medicaid, or uninsured	47 (65.3)	351 (60.3)	1.24 (0.74 to 2.07)	98 (63.2)	300 (60.1)	1.14 (0.79 to 1.67)
Low SES^d^	9 (12.5)	48 (8.2)	1.59 (0.75 to 3.39)	16 (10.3)	41 (8.2)	1.29 (0.70 to 2.36)
Infant sex, male	43 (56.6)	333 (55.6)	1.02 (0.62 to 1.66)	87 (55.8)	280 (55.8)	1.00 (0.70 to 1.44)
Chronic lung disease	53 (69.7)	287 (47.9)	2.75 (1.62 to 4.69)	101 (64.7)	228 (45.4)	2.21 (1.52 to 3.20)
Severe retinopathy	9 (11.8)	31 (5.2)	2.60 (1.18 to 5.72)	18 (11.5)	21 (4.2)	2.99 (1.55 to 5.77)
Any neonatal sepsis	14 (18.4)	73 (12.2)	1.57 (0.82 to 3.00)	28 (17.9)	56 (11.2)	1.74 (1.06 to 2.86)
Outborn status	28 (36.8)	105 (17.5)	2.69 (1.58 to 4.57)	40 (25.6)	80 (15.9)	1.82 (1.12 to 2.80)
PMA at birth, mean (SD), wk	26.3 (2.04)	27.1 (1.88)	Mean difference, 0.830 (95% CI, 0.365 to 1.294)	26.4 (2.02)	27.3 (1.84)	Mean difference, 0.902 (95% CI, 0.563 to 1.241)
PMA at NNNS examination, mean (SD), wk	41.4 (3.58)	39.0 (3.31)	Mean difference, −2.380 (95% CI, −3.189 to 1.571)	40.1 (3.49)	39.0 (3.33)	Mean difference, −1.054 (95% CI, −1.661 to 0.447)
Fetal growth restriction	5 (6.8)	45 (7.7)	0.88 (0.34 to 2.30)	8 (5.2)	42 (8.4)	0.60 (0.27 to 1.30)
Birthweight, g	897 (287)	962 (281)	Mean difference, 65.455 (95% CI, −3.204 to 134.115)	915 (290)	967 (278)	Mean difference, 52.549 (95% CI, 1.752 to 103.35)
Head circumference, mean (SD), cm	23.8 (2.32)	24.6 (2.4)	Mean difference, 0.731 (95% CI, 0.133 to 1.328)	24.2 (2.61)	24.6 (2.38)	Mean difference, 0.419 (95% CI, −0.024 to 0.886)

Abbreviations: CUS, cranial ultrasound; NNNS, NICU Network Neurobehavioral Scale; OR, odds ratio; PMA, postmenstrual age; SES, socioeconomic status.

^a^ White matter damage included periventricular leukomalacia, moderate to severe ventriculomegaly, and echolucency.

^b^ Early CUS lesions included germinal matrix hemorrhage, moderate to severe ventriculomegaly, intraventricular hemorrhage, and parenchymal echodensity.

^c^ Maternal reported minority race or ethnic group of American Indian, Asian, Black, Hispanic, Native Hawaiian, Pacific Islander, or other specified by mother.

^d^ SES was calculated using the 4-factor Hollingshead Index (based on marital status, employment status, educational attainment, and occupation), which has been adapted to single-parent and non–nuclear families, with Hollingshead level V indicating low SES.

### Illness and Neurobehavior Associated With Ultrasound Indicators of WMD

Infants with ultrasound indicators of WMD, compared with infants without these indicators, were more likely to develop CLD (53 of 76 [69.7%] vs 287 of 599 [47.9%]) and severe ROP (9 [11.8%] vs 31 [5.2%]). They were more likely to be outborn (28 [36.8%] vs 105 [17.5%]), born earlier (mean [SD] PMA at birth, 26.3 [2.0] weeks vs 27.1 [1.9] weeks), and undergo the NNNS at an older mean (SD) PMA (41.4 [3.6] weeks vs 39.0 [3.3] weeks). (Table 1). NNNS examination revealed infants with WMD had lower attention scores, higher excitability, were hypotonic, with more asymmetric reflexes, and poorer quality of movement (Table 2 and eTable in the Supplement). Associations between WMD and lower attention scores (adjusted mean difference, −0.346; 95% CI, −0.609 to −0.083), hypotonicity (adjusted mean difference, 0.358; 95% CI, 0.055 to 0.662), and poorer quality of movement (adjusted mean difference, −0.344; 95% CI, −0.572 to −0.116) persisted after adjusting for infant gender, CLD, severe ROP, any sepsis, minority race or ethnicity, outborn vs inborn status, PMA at birth and NNNS exam, and study site (Table 3).

**Table 2.** Neonatal Intensive Care Unit Network Neurobehavioral Scale Summary Scores by White Matter Damage and Early CUS Lesions

Summary score	Mean (SD)		Mean difference (95% CI)	Mean (SD)		Mean difference (95% CI)
	White matter damage (n = 76)^a^	No white matter damage (n = 599)		Early CUS lesions (n = 156)^b^	No early CUS lesions (n = 502)	
Attention	4.88 (1.64)	5.34 (1.47)	0.489 (0.123 to 0.855)	4.91 (1.56)	5.38 (1.44)	0.467 (0.197 to 0.737)
Handling	0.46 (0.26)	0.40 (0.27)	−0.049 (−0.114 to 0.016)	0.42 (0.26)	0.40 (0.27)	−0.021 (−0.070 to 0.026)
Self-regulation	5.53 (0.86)	5.63 (0.79)	0.113 (−0.081 to 0.307)	5.61 (0.79)	5.63 (0.80)	0.023 (−0.120 to 0.166)
Arousal	3.81 (0.82)	3.74 (0.62)	−0.065 (−0.223 to 0.093)	3.73 (0.71)	3.74 (0.63)	0.011 (−0.105 to 0.128)
Excitability	3.00 (2.39)	2.41 (2.00)	−0.564 (−1.063 to −0.065)	2.50 (2.16)	2.44 (2.02)	−0.056 (−0.425 to 0.314)
Lethargy	4.88 (2.49)	4.50 (2.06)	−0.448 (−0.960 to 0.065)	4.92 (2.36)	4.48 (2.01)	−0.439 (−0.817 to −0.061)
Hypertonicity	0.45 (0.84)	0.37 (0.73)	−0.025 (−0.201 to 0.151)	0.31 (0.67)	0.38 (0.74)	0.071 (−0.059 to 0.201)
Hypotonicity	0.34 (0.78)	0.21 (0.46)	−0.130 (−0.254 to −0.007)	0.33 (0.67)	0.19 (0.44)	−0.140 (−0.231 to −0.049)
Nonoptimal reflexes	5.67 (2.03)	5.30 (2.07)	−0.376 (−0.878 to 0.127)	5.36 (1.94)	5.35 (2.10)	−0.010 (−0.382 to 0.361)
Asymmetric reflexes	1.32 (1.45)	0.94 (1.27)	−0.310 (−0.619 to −0.001)	1.01 (1.33)	0.94 (1.26)	−0.073 (−0.302 to −0.156)
Quality of movement	4.43 (0.69)	4.61 (0.69)	0.179 (0.012 to 0.347)	4.54 (0.70)	4.61 (0.69)	0.069 (−0.055 to 0.193)
Stress abstinence	0.14 (0.08)	0.14 (0.07)	0.003 (−0.015 to 0.020)	0.14 (0.07)	0.14 (0.07)	0.003 (−0.013 to 0.013)

Abbreviation: CUS, cranial ultrasound.

^a^ White matter damage included periventricular leukomalacia, moderate to severe ventriculomegaly, and echolucency.

^b^ Early CUS lesions included germinal matrix hemorrhage, moderate to severe ventriculomegaly, intraventricular hemorrhage, and parenchymal echodensity.

**Table 3.** White Matter Damage and Risk for Poor Neurodevelopmental Outcomes

NNNS summary score	Adjusted mean difference (95% CI)^a^
Attention	−0.346 (−0.609 to −0.083)
Excitability	0.228 (−0.040 to 0.495)
Hypotonicity	0.358 (0.055 to 0.662)
Asymmetric reflexes	0.198 (−0.056 to 0.452)
Quality of movement	−0.344 (−0.572 to −0.116)

Abbreviation: NNNS, NICU Network Neurobehavioral Scale.

^a^ Adjusted models include infant sex, chronic lung disease, severe retinopathy of prematurity, any sepsis, maternal minority race or ethnicity, outborn vs inborn status, postmenstrual age at birth, postmenstrual age at NNNS examination, and study site.

### Illness and Neurobehavior Associated With CUS Lesions Detected on Early Scans

Infants with CUS lesions (GMH, moderate to severe VM, IVH, and PED), as compared with infants without such lesions, were more likely to have developed CLD (101 of 156 [64.7%] vs 228 of 502 [45.4%]), severe ROP (18 [11.5%] vs 21 [4.2%]), and neonatal sepsis (28 [17.9%] vs 56 [11.2%]). They were more likely to be outborn (40 [25.6%] vs 80 [15.9%]), born earlier (mean [SD] PMA age at birth, 26.4 [2.0] weeks vs 27.3 [1.8] weeks), and undergo NNS at an older mean (SD) PMA (40.1 [3.5] weeks vs 39.0 [3.3] weeks) (Table 1). Infants with early CUS lesions had lower attention scores, were more lethargic, and were more hypotonic (Table 2). The associations with attention scores (adjusted mean difference, −0.233; 95% CI, −0.423 to −0.044) and hypotonicity (adjusted mean difference, 0.240; 95% CI, 0.014 to 0.465) persisted after adjusting for infant gender, CLD, severe ROP, any sepsis, minority race or ethnicity, outborn vs inborn status, PMA at birth and NNNS examination, and study site (Table 4).

**Table 4.** Early Cranial Ultrasound Lesions and Risk for Poor Neurodevelopmental Outcomes

NNNS Summary Score	Adjusted mean difference (95% CI)^a^
Attention	−0.233 (−0.423 to −0.044)
Lethargy	0.153 (−0.046 to 0.352)
Hypotonicity	0.240 (0.014 to 0.465)

Abbreviation: NNNS, NICU Network Neurobehavioral Scale.

^a^ Adjusted models include infant sex, chronic lung disease, severe retinopathy of prematurity, any sepsis, maternal minority race or ethnicity, outborn vs inborn status, postmenstrual age at birth, postmenstrual age at NNNS examination, and study site.

Based on the approach to interpretation of effect sizes suggested by Cohen,^27^ effect sizes for association between WMD and hypotonicity and poor quality of movement fell in the medium effect size range (0.61 and 0.44, respectively) and in the small effect size range for attention (0.25). For early lesions and hypotonicity, the effect size fell in the medium range for hypotoncity (0.39) and in the small range for attention (0.17).

## Discussion

We examined associations between CUS findings and neurobehavior assessed using the NNNS, toward the goal of detecting early risk for poorly regulated neonatal attention, arousal, tone, and movement, and to identify need for intervention in the NICU and beyond. To our knowledge, this is the first study to examine the association between CUS findings obtained in very low-birth-weight infants and NNNS neurobehavioral outcomes conducted at NICU discharge between 34 and 51 weeks’ PMA.

In this sample of infants born before 30 weeks’ gestation, abnormal findings on CUS performed in the first 2 weeks of life were associated with less optimal attention scores and hypotonicity in the week prior to discharge from neonatal intensive care. These neurobehavioral outcomes, as well as poor quality of movement, were associated with ultrasound indicators of WMD, identified on CUS performed after the first postnatal month. The effect sizes reported here for associations between ultrasound lesions and neurobehavioral outcomes are comparable with those found in a previously reported cohort of premature infants born at 24 to 32 weeks’ gestational age.^25^ Effect size is an important indicator of clinical significance reflecting the magnitude of differences between 2 groups. Clinically significant effect sizes have implications for intervention and small effect sizes can indicate public health significance.^28^

Earlier small, single-site studies of neonatal CUS and neurobehavior in very preterm infants (<32 weeks’ PMA) found similar associations between neonatal CUS and motor outcomes at term. In 1 study of very preterm infants with fetal growth restriction, an association between increased ventricular volume on CUS and impaired performance on Tests of Infant Motor Performance at 4 to 6 weeks and 12 to 14 weeks’ postnatal age was reported.^29^ Additionally, in another study, quantitative measurement of periventricular white matter echogenicity was associated with neuromotor status at term- or near term-age as measured by the Lacey Assessment of the Preterm Infant, but early CUS findings were not associated with performance on this examination.^30^

This consistency in short-term associations is comparable with associations between the NNNS and structural volumes on magnetic resonance imaging (MRI)^10,16^ and electroencephalogram findings.^18,31^ These collective findings support the use of more proximal assessments of brain structure and function to identify early threats to subsequent neurodevelopment and to potentially inform NICU and postdischarge care planning.

Prediction of neurodevelopment through childhood is important to both clinicians and families. Our findings suggest that developmental interventions in the NICU might be appropriate for infants with structural abnormalities identified as being associated with adverse neurobehavioral outcome at NICU discharge. The associations between WMD and NNNS scores for attention, movement, and tone might constitute an early phase in the putative link between ultrasound-identified WMD and later cerebral palsy as well as cognitive and behavioral impairments.^32,33^

The association between CUS and neurodevelopmental outcome has been well studied. Echolucent lesions and VM on CUS are associated with delayed mental and psychomotor development at 24 months’ corrected age.^3^ Additional studies continue to support the association between WMD and later neurodevelopmental impairments, including cognitive and gross motor impairment.^34,35^ The importance of early CUS findings is suggested by the finding that cystic PVL, which seems to disappear on serial ultrasound scans, is associated with increased risk of adverse outcome.^36^ Although MRI is more sensitive than CUS for detection of WMD, CUS is safer and more efficient (ie, can be accomplished without moving the infant out of the NICU) and significantly less expensive than MRI. Furthermore, near-term conventional MRI is not superior to CUS in predicting severe impairments at early school-age.^37^

The NNNS has demonstrated reliability and validity in the prediction of early childhood outcomes of premature and other high risk infants.^14,33^ In very preterm infants with severe IVH and VM,^38^ nonoptimal reflexes on the NNNS examination was associated with lower motor, cognitive, and language scores. Less regulation and more nonoptimal reflexes have been associated with lower Bayley Mental Development Index scores, and poor regulation, more nonoptimal reflexes, hypertonicity, and increased handling needs have been associated with lower Psychomotor Development Index scores.^18^ In a study of children exposed to prenatal cocaine and/or other substances, NNNS risk profiles at 1 month of age predicted behavior problems, school readiness, and IQ through 4.5 years of age,^14^ and NNNS scores reflecting poor arousal and stress responses predicted behavioral problems at age 3 and 7 years.^15^ Additionally, neonatal neurobehavioral testing in preterm infants has been associated with positive screening for autism at 2 years and sensory processing disorders in children 4 to 6 years of age.^16,39^

Early CUS lesions have the potential to identify infants who might benefit from interventions to reduce or prevent hypotonicity and to improve movement quality and attention. Potential strategies include (1) closer monitoring by therapists for infants with early CUS and WMD insults and (2) providing enriched environments with supportive interventions to focus on calming motoric agitation as well as improving relaxed alertness, balanced motor tone, involuntary and coordinated movements to facilitate sustained focused attention, arousal regulation, and improve hypotonicity and movement quality.

### Strengths and Limitations

Strengths of this study include the large multicenter sample size; high reliability of CUS findings derived from consensus readings by at least 2 masked, centralized readers; and standardized, validated neurobehavioral assessments by examiners masked to infant medical history. Additionally, it is the first known study examining the association between ultrasound and this early indicator of neurodevelopment.

This study has limitations, including our failure to obtain ultrasound images through the mastoid window, which improves detected of cerebellar abnormalities. Furthermore, a heterogeneous group of infants with regards to stability and survivability was included.

## Conclusions

In this cohort study of preterm infants, early CUS lesions and WMD were associated with poor regulation of attention, tone, and movement outcomes measured by an NNNS examination at NICU discharge. The specific NNNS risks identified here are characteristics that are associated with neurodevelopmental impairments throughout early childhood. Addressing demonstrated risk with targeted prevention efforts in the NICU and postdischarge referrals has the potential to improve long-term outcomes.

## Notes

Supplement. **eTable.** White Matter Damage, Early Cranial Ultrasound Lesions, and Risk for Poor Neurodevelopmental Outcomes
