# Supplementary material for: Association of Abnormal Findings on Neonatal Cranial Ultrasound With Neurobehavior at Neonatal Intensive Care Unit Discharge in Infants Born Before 30 Weeks’ Gestation
Source: JAMA Netw Open. 2022 Apr 8;5(4):e226561. doi: 10.1001/jamanetworkopen.2022.6561 (PMC8994127; doi:10.1001/jamanetworkopen.2022.6561)
Supplement: Supplement. — eTable. White Matter Damage, Early Cranial Ultrasound Lesions, and Risk for Poor Neurodevelopmental Outcomes [file jamanetwopen-e226561-s001.pdf]

Supplemental Online Content

Helderman J, O'Shea TM, Dansereau L, et al. Association of abnormal findings on neonatal cranial ultrasound with neurobehavior at neonatal intensive care unit discharge in infants born before 30 weeks' gestation. *JAMA Netw Open.* 2022;5(4):e226561. doi:10.1001/jamanetworkopen.2022.6561

eTable. White Matter Damage, Early Cranial Ultrasound Lesions, and Risk for Poor Neurodevelopmental Outcomes

This supplemental material has been provided by the authors to give readers additional information about their work.

© 2022 Helderman J et al. *JAMA Network Open.*

eTable. White Matter Damage, Early Cranial Ultrasound Lesions, and Risk for Poor Neurodevelopmental Outcomes

NNNS summary score	Adjusted Mean Difference*	95% CI
**White Matter Damage**		
Attention	-0.346	-0.609, -0.083
Handling	0.271	-0.012, 0.520
Self-Regulation	-0.091	-0.332, 0.150
Arousal	0.081	-0.227, 0.389
Excitability	0.228	-0.040, 0.495
Lethargy	0.224	-0.053, 0.500
Hypertonicity	0.003	-0.248, 0.254
Hypotonicity	0.358	0.055, 0.662
Non-optimal Reflexes	0.128	-0.118, 0.375
Asymmetric Reflexes	0.198	-0.056, 0.452
Quality of Movement	-0.344	-0.572, -0.116
Stress Abstinence	0.084	-0.170, 0.337
**Early Lesions**		
Attention	-0.233	-0.423, -0.044
Handling	0.164	-0.018, 0.346
Self-Regulation	0.028	-0.148, 0.205
Arousal	-0.001	-0.197, 0.195
Excitability	0.034	-0.150, 0.217
Lethargy	0.153	-0.046, 0.352
Hypertonicity	-0.086	-0.256, 0.083
Hypotonicity	0.235	0.031, 0.438
Non-optimal Reflexes	-0.074	-0.248, 0.100
Asymmetric Reflexes	0.058	-0.122, 0.238
Quality of Movement	-0.182	-0.359, -0.006
Stress Abstinence	0.078	-0.096, 0.251

*Adjusted models include infant sex, CLD, severe ROP, any sepsis, minority race or ethnicity, outborn vs inborn status, PMA at birth, PMA at NNNS exam and study site.

© 2022 Helderman J et al. *JAMA Network Open.*
